# Flexible feature mapping topology optimization using NURBS-based component projection

**DOI:** 10.1007/s00158-025-04047-2

**Published:** 2025-07-09

**Authors:** Reinier Giele, Can Ayas, Matthijs Langelaar

**Affiliations:** Faculty of Mechanical Engineering, Delft University of Technology, Mekelweg 2, 2628 CD Delft, The Netherlands

**Keywords:** Topology optimization, Geometry, Accessibility, Milling, Cleaning, Molding

## Abstract

A novel feature mapping topology optimization method is presented, allowing for the creation of features with highly flexible shapes. The method easily integrates with conventional density-based formulations. Feature shapes are implicitly described by NURBS control points. The feature shape dictates the locations of two sets of projection points to represent the solid void boundaries. At these projection points, density values are projected onto a finite element mesh. The method optimizes feature shapes in a gradient-based manner, while allowing more specific control of the feature shapes than classical level set methods. Several feature fields can be combined to create a final output design. It is found that the eminent flexibility of the NURBS-based feature definition is a benefit but also requires additional regularization to guarantee stability of the optimization.

## Introduction

Topology optimization (TO) is a computational design method to determine the distribution of material, such that the geometric layouts of components with superior performance can be determined. Several distinct TO approaches exist, where the most prevalent ones are density-based methods (see e.g., Sigmund and Maute 2013) and level-set (LS) methods (see e.g., Van Dijk et al. 2013). Density methods focus on the distribution of material (represented by a density field) in a discretized domain, benefiting from a of lack of restrictions on design changes throughout the design domain, while sensitivity information is available everywhere in the design domain. In LS methods the design is generated by moving the boundary between solid and void regions, benefiting from having a clear boundary description.

Another more recent category of TO approaches is known as feature mapping methods (FMMs), see Wein et al. (2020) for an overview. In FMMs, solid (or void) features with specified geometric shapes are usually mapped onto a finite element mesh. While the shape of the features is often fixed, typically with limited options such as (hyper) ellipses or rectangles in 2D and their counterparts in 3D (Wein et al. 2020), their position, orientation, and size are optimized. The moving feature boundaries bear similarities to LS methods with a more strict shape restriction of each feature, such that simpler design outcomes are ensured. Final component designs are often obtained through combining and overlapping these features.

Several challenges occur in creating components by combining features. First, there is limited to no control over the final topology. Since designs in current FMMs are typically created with many features, the topology can easily change. For specific applications topology control can be required or desired, see e.g., He et al. (2023).

Secondly, for designs created with fewer features, the design space is limited. One way to allow for complex designs with fewer components is to allow some flexibility in the feature shape. In the method of moving morphable components (MMC) framework, this has been addressed, e.g., by Shannon et al. (2022) with curved features with varying thickness defined using generalized Bézier curves, or by Zheng and Kim (2020) with NURBS (non-uniform rational basis splines) shaped components (although only one fixed feature shape is considered). To the best of our knowledge, the feature shapes in the aforementioned studies are almost unchangeable, limiting the design freedom of the resulting layout. An exception is, e.g., Zhang et al. (2017) the shape of features is optimized with closed B-splines, however only limited shape flexibility is allowed in this parameterization. Other examples of limited spline based TO include Greifenstein et al. (2023), Guo et al. (2016), and Zhu et al. (2021).

Thirdly, it is beneficial if the topology optimized design geometry can easily be converted to a common CAD (computer aided design) description, which is often required for further processing of the design. For this purpose, TO methods have been proposed that are focused on explicit output shapes. For instance, Schmidt et al. (2023) consider a semi-analytical gradient-based optimization of exact CAD models using intermediate field representations, and Yi and Kim (2017) identify the boundaries of TO results to extract basic parametric features to close the gap with parameterized CAD models.

To address the three aforementioned gaps simultaneously, this paper studies and presents a novel FMM framework for creating features with flexible shapes using a NURBS parameterization, such that feature shapes are adjustable during the optimization for more design freedom and convenient for post-processing of designs.

**Fig. 1:**
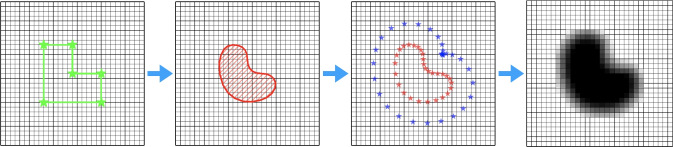
A schematic illustration of the method, for creating one feature in a 2D design domain. First, design variables (*control points* location and weights) represented by green stars are used to create a feature shape. Next, the feature shape is discretized, and two sets of *projection points* (represented by red and blue stars) are defined. Lastly, these two sets of projection points are projected onto the finite element mesh to create an output density field

The steps of the proposed method starting from design variables to a feature geometry are illustrated in Fig. 1. First, we define the parameterization of the feature’s boundary shape. We opt to use the locations and weights of the *control points* (CPs) as design variables. Next, the discretized feature boundary curve defines the first set of *projection points* (PPs), whereas a second set is of PPs is located at an offset outside the feature boundary. These two sets of PPs are used to project a solid, respectively, void density value onto the the underlying mesh, eventually creating an aggregate output density field. This use of projection points to relate the NURBS curve to the density field is the main novel concept we introduce, and allows for a convenient integration with conventional density-based TO procedures. The performance of the design projected onto the mesh is obtained through finite element analysis, and using a gradient-based TO process, the design variables are optimized iteratively, so that the feature shapes are adapted toward the optimal geometry.

An important benefit of the proposed method is that intricate shapes can be attained by each feature, such that complex output designs can be created, even when using relatively few features. Better control over the topology is achievable, and an easier conversion to a useful output design is facilitated through the use of the NURBS description. The density field output allows the proposed novel method to build on the existing standard density-based TO, and even can be combined with standard density fields, where both the standard density field and the feature associated density field together describe the component. Since the design variables, i.e., the CP locations and weights, are mathematically related to element densities, gradient-based optimization can be used with conventional density-based adjoint sensitivity analysis, building on density-based TO implementations. Naturally, the method can be applied both to solid and void features. An opacity design variable is added to allow for features to disappear [similar to Norato et al. (2015)].

This paper focuses on the 2D version of the new method, both for clarity and because it already presents a considerable number of novel aspects to investigate. An extension to 3D is out of the scope of the current study, but the extension of the method for 3D cases is discussed in Sect. 4. Finally, it should be mentioned that the proposed method also poses new challenges related to shape restriction, therefore extra shape regularization schemes are extensively outlined in this study.

## Method

For a simple and clear description of the method, solid features are considered within a design domain discretized with a structured mesh. Section 2.1 presents the procedure for creating NURBS shapes. Section 2.2 describes how the NURBS shapes are projected onto the mesh as a density field. Lastly, several regularization schemes are proposed in Sect. 2.3 to improve the optimization stability.

### NURBS with continuous shape functions

**Fig. 2:**
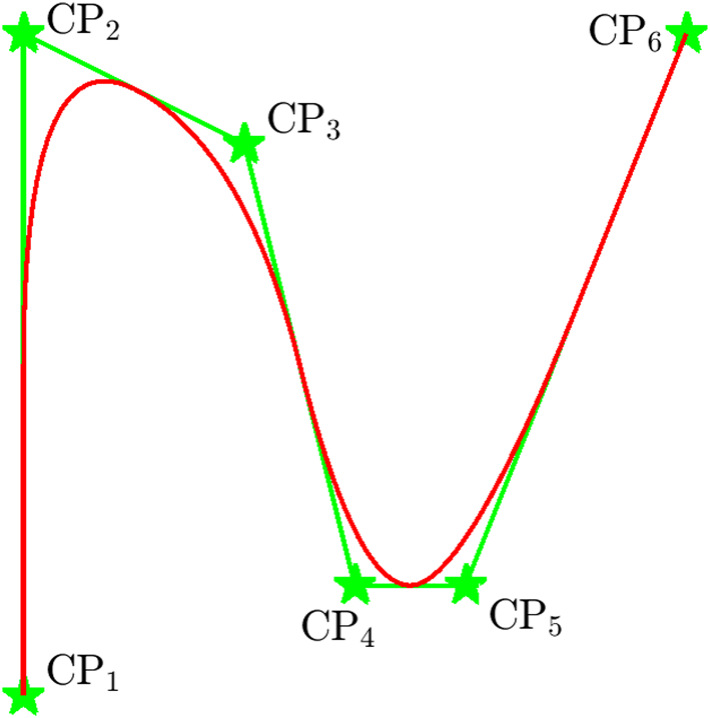
An example of an open NURBS curve (red) created from six CPs (green stars)

**Fig. 3:**
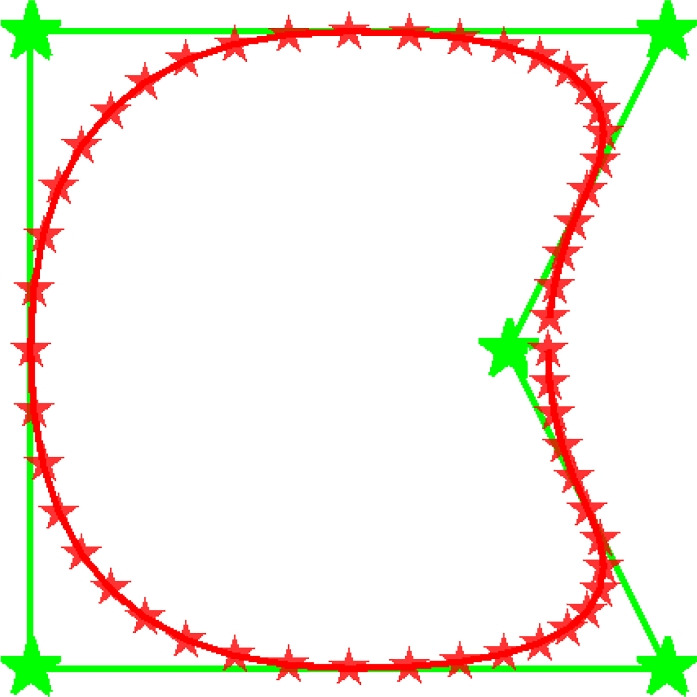
A closed boundary curve defining the outline of a feature (red stars), created from five CPs (green stars)

This section describes the steps to obtain feature shapes from NURBS *control points* (CPs), defined by design variables. NURBS are a generalization of B-splines, while B-splines are a generalized form of the Bézier curve. NURBS represent shapes with great versatility, an example of an open NURBS curve is shown in Fig. 2, and an example of a discretized NURBS curve is shown in Fig. 3. For more background information on NURBS, the reader is referred to, e.g., Piegl and Tiller (1997) and Cottrell et al. (2009).

As outlined in the introduction, the proposed method defines feature shapes using CPs. Each CP has a specified local influence on the boundary curve. The CP locations and weights, together with appropriate basis functions, can describe a complex shape. As design variables, we introduce the arrays $\textbf{p}^{\text {(x)}}$, $\textbf{p}^{\text {(y)}}$, and $\textbf{p}^{\text {(w)}}$, which represent *x*- and *y*-coordinates of the CPs and their weights, respectively.

First, the knot vector is the sequence of parameter values that determine where and how the control points affect the NURBS curve. In this work, the knot vector is defined as $\mathcal {U} = \left[ u_{1},..., u_{m} \right]$, which is a non-decreasing sequence of real numbers. Here, $u_{i}$ are the knots, where $i=1,\ldots ,m$ is the knot index, and the knot vector has length $m=k+q+1$, where *q* is the polynomial order of the basis functions, and *k* is the number of basis functions used to construct the NURBS curve. In our application, we will use an unclamped, uniform, knot vector, which means that all knot spans are of equal length, e.g., $\mathcal {U} = \left[ 0,1,2,...,8,9,10\right]$.

Next, the zeroth degree ($q=0$) basis function $N_{i,0}$ is described as a piece-wise function of curve parameter *u*:

1 $$\begin{aligned} N_{i,0}(u)= {\left\{ \begin{array}{ll} 1 & \text {if} \quad u_{i} \le u < u_{i+1}, \\ 0 & \text {otherwise}. \end{array}\right. } \end{aligned}$$

The higher ($q>0$) degree basis functions $N_{i,q}$ are calculated by recursion:

2 $$\begin{aligned} \begin{aligned} N_{i,q}(u) =&\frac{ u-u_{i} }{ u_{i+q}-u_{i} } N_{i,q-1} (u) + \\&\frac{ u_{i+q+1}-u }{ u_{i+q+1}-u_{i+1} } N_{i+1,q-1} (u). \end{aligned} \end{aligned}$$

In our application, second order ($q=2$) basis functions are used.

With the basis functions $N_{i,q}$, the weight of each control point ${p}_{i}^{\text {(w)}}$ and its *x*-coordinates ${p}_{i}^{\text {(x)}}$, the NURBS curve *x*-coordinates, ${c}^{\text {(x)}} (u)$, are calculated as follows:

3 $$\begin{aligned} {c}^{\text {(x)}} (u) = \frac{ \sum ^{k}_{i=1} N_{i,q} (u) {p}_{i}^{\text {(w)}} {p}_{i}^{\text {(x)}} }{ \sum ^{k}_{i=1} N_{i,q} (u) {p}_{i}^{\text {(w)}} } . \end{aligned}$$

The coordinates in other dimensions (e.g., ${c}^{\text {(y)}} (u)$) follow similarly. Finally, we note that assuming a knot vector beforehand restricts feature shapes. Sharp corners for example require repeated knot values. In this study, we do not allow repeated knot values and consequently we restrict ourselves to features without intrinsic sharp corners.

**Fig. 4:**
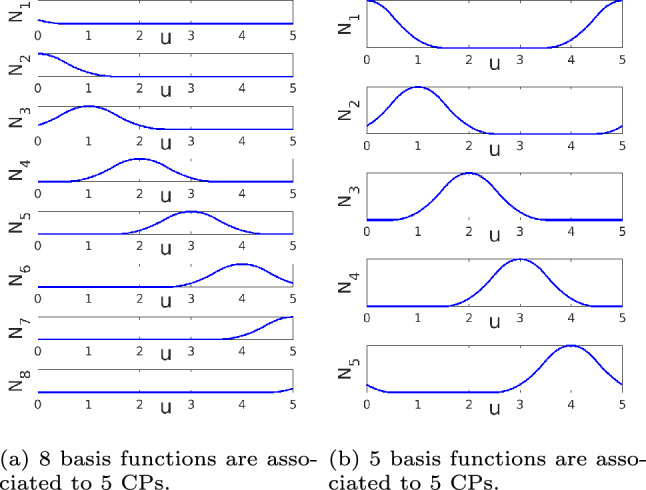
An example of second order basis functions with five CPs. **a** $\hbox {CP}_{1}$ is associated to $N_{2}$ and $N_{7}$, $\hbox {CP}_{2}$ is associated to $N_{3}$ and $N_{8}$, and $\hbox {CP}_{3}$ is associated to $N_{1}$ and $N_{6}$. **b** The basis functions for the 1 st, 2nd, and 3rd basis functions are combined with the 6 th, 7 th, and 8 th basis functions, respectively

Finally, since the feature shape is defined by a closed boundary curve that encloses a finite area, we opt for the curve to be second order continuous at the start, which also coincides with the end of the curve. This is implemented by incorporating three extra CPs, with locations and weights that are identical to the first three CPs. For example, for a 5 CP feature, ${p}^{\text {(x)}}_{1}={p}^{\text {(x)}}_{6}$, ${p}^{\text {(x)}}_{2}={p}^{\text {(x)}}_{7}$, and ${p}^{\text {(x)}}_{3}={p}^{\text {(x)}}_{8}$, and the same holds for $\textbf{p}^{\text {(y)}}$ and $\textbf{p}^{\text {(w)}}$. An example of the basis functions is shown in Fig. 4a, and the basis function for each unique CP is shown in Fig. 4b. The reader is referred to Piegl and Tiller (1997) for more examples.

### Creating features with projection points

**Fig. 5:**
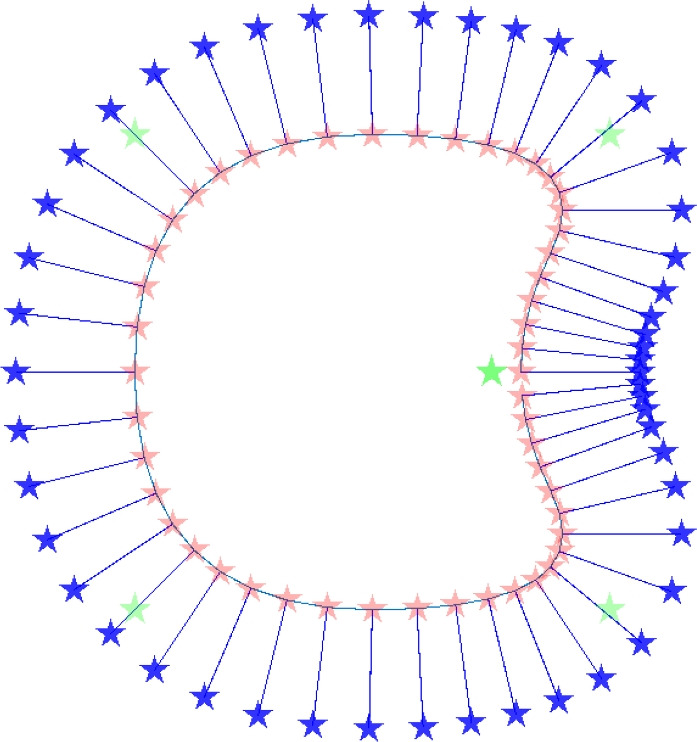
An example of the two sets of PPs, with $n^{\text {(CP)}}=10$. The solid PPs (in red) are located on the curve depicted in Fig. 3. The void PPs (in blue) lie on an offset curve with respect to the solid PP curve

It remains to describe the steps to obtain a density field from a NURBS curve. First, the continuous NURBS curve is discretized. In our application, this is done by discretizing the basis functions $N_{i,q}$, with $n^{\text {(CP)}}$ points per CP, resulting in a discretized NURBS curve. Next, two sets of *projection points* (PPs) are defined: *‘solid PPs’* which are located on the feature’s surface, and *‘void PPs’* located outside of the feature. Consequently, in our application, the solid PP location, $\mathbf {\dot{c}}_{j}$, coincides with the discretized NURBS boundary curve described in Eq. (3), where integer *j* denotes the discretized PP index. The void PPs, each denoted with $\mathbf {\mathring{c}}_{j}$, are located at an offset curve with respect to $\mathbf {\dot{c}}_{j}$:

4 $$\begin{aligned} \mathbf {\mathring{c}}_{j} = \mathbf {\dot{c}}_{j} + r \textbf{n}_{j}, \end{aligned}$$

where *r* is the offset distance. The calculation of unit outward normal $\textbf{n}_{j}$ is given in Sect. 2.3. An example of the calculated void PPs is shown in Fig. 5.

**Fig. 6:**
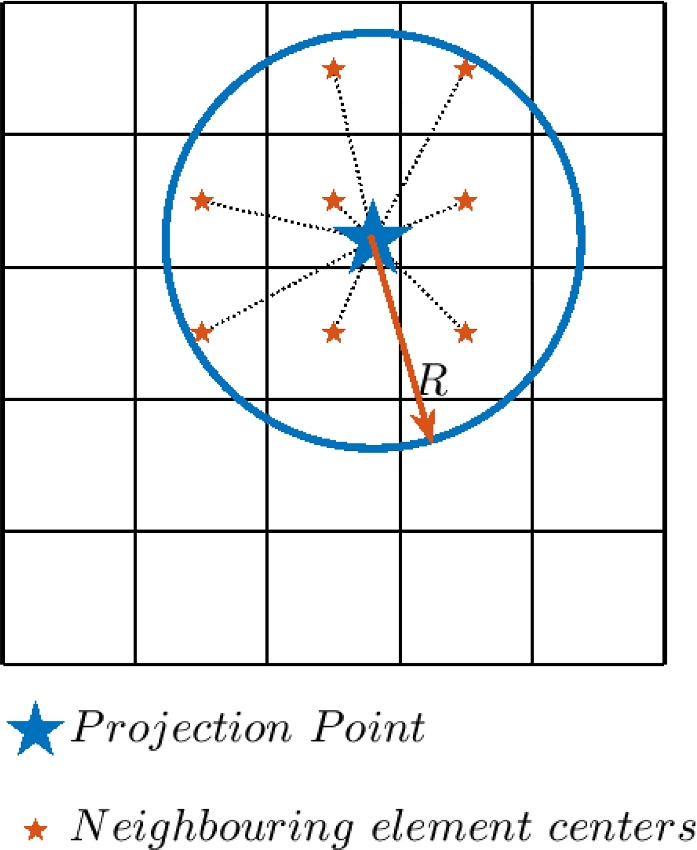
Projection points are used to project a density value onto its surrounding elements

Once all PP coordinates have been calculated using Eqs. (3) and (4), the feature density field can be created. Each PP projects a specified density value, $\chi$, onto several fixed mesh elements in its close proximity. The density projection is performed similar to the standard convolution filter (Bourdin 2001; Bruns and Tortorelli 2001), as shown in Fig. 6. However, note that since the PPs are detached from the mesh, they are not restricted to coincide with element centers.

The spatial weights associated with the projection to each element are calculated using the proximity of the element center $\textbf{c}_{e}$ to PP *j* as follows:

5 $$\begin{aligned} w_{e,j}(\textbf{c}_{j}) = \text {max} \left( 0 , R - \Vert \textbf{c}_{j} - \textbf{c}_{e} \Vert \right) . \end{aligned}$$

Here, *R* is the interpolation radius, and $\textbf{c}_{j}$ and $\textbf{c}_{e}$ are the coordinates of the PP and the centroid of element *e*, respectively. $\textbf{c}_{j}$ represents both solid ($\mathbf {\dot{c}}_{j}$) and void ($\mathbf {\mathring{c}}_{j}$) PPs. The calculation of $w_{e,j}$ ensures a gradual transition from solid to void at the feature boundary, which allows for sensitivity analysis. Note that these weights are related to the density projection, and are not linked to the CP weights defining the NURBS curve, which are controlled by the optimizer. Also note that the subscript *e*, *j* refers to element *e* and PP *j*, and the comma in the subscripts does not denote differentiation in this paper. These interpolation weights are subsequently normalized as follows:

6 $$\begin{aligned} \check{w}_{e,j} (\textbf{c}_{j}) = \frac{ w_{e,j} (\textbf{c}_{j}) }{ \sum ^{N_{\text {el}}}_{e=1} w_{e,j} (\textbf{c}_{j}) }, \end{aligned}$$

where $N_{\text {el}}$ is the number of elements in the domain. Next, the output value $\chi$ is projected to element *e* resulting in a projected value:

7 $$\begin{aligned} \check{x}_{e,j} = \chi \check{w}_{e,j}. \end{aligned}$$

The solid PPs each project a value of $\chi =1$, the void PPs each project a value of $\chi =0$. To account for elements getting contributions from multiple PPs, the final output value for element *e* is obtained by dividing the sum of the projected values by the sum of the normalized weights of the contributions:

8 $$\begin{aligned} \bar{x}_{e} = \frac{ \sum ^{{n}_{j}}_{j=1} \check{x}_{e,j} }{ \sum ^{{n}_{j}}_{j=1} \check{w}_{e,j} }, \end{aligned}$$

where $n_{j}$ is the number of PPs that has projected a (solid or void) density to element *e*, and $\check{x}_{e,j}$ and $\check{w}_{e,j}$ are the value and weight of the individual contributions. Note that this operation with only one nonzero weight contribution results in $\bar{x}_{e}=\chi$, thus taking exactly the density value from the only contributing PP.

**Fig. 7:**
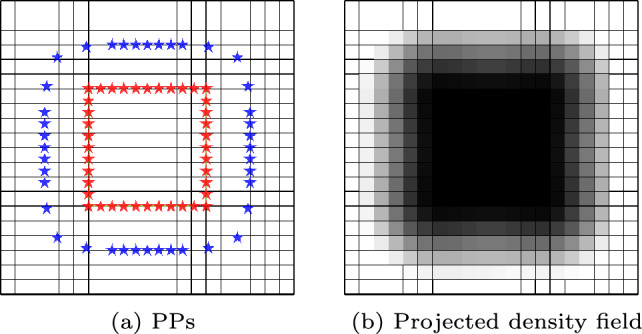
Solid (red) and void (blue) projection points are used to project density onto the structured mesh, representing a feature. The intermediate density region between the solid and void PPs ensures consistent sensitivities for the feature boundary location

An example of solid (red) and void (blue) PPs, and the resulting density field projected onto the FE mesh is shown in Fig. 7. The elements inside the feature, where the element centroid is not close enough to any PPs are assigned a density value of $\bar{x}_{e}=1$. The intermediate density zone across the solid-void interface enables obtaining a differentiable boundary motion and is hence required to calculate consistent sensitivities for the feature’s boundary shape and location.

**Fig. 8:**
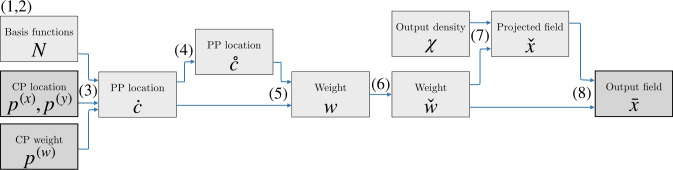
The flow diagram illustration of the steps from the PP locations and weights to the feature density field. The numbers represents the equations used for each step. By following the steps backwards, and through the chain rule, the sensitivities follow naturally

The equations used for the steps from the PP locations to the output density field $\mathbf {\bar{x}}$ are shown schematically in Fig. 8. The sensitivities ${\partial \mathbf {\bar{x}} }/{\partial \textbf{p}^{\text {(x)}} }$, ${\partial \mathbf {\bar{x}} }/{\partial \textbf{p}^{\text {(y)}} }$, and ${\partial \mathbf {\bar{x}} }/{\partial \textbf{p}^{\text {(w)}} }$ follow naturally with the chain rule for each operation in Fig. 8, such that the sensitivities of the objective/constraints with respect to $\textbf{p}^{\text {(x)}}$, $\textbf{p}^{\text {(y)}}$, and $\textbf{p}^{\text {(w)}}$ can be computed. The integration of specific constraints is thus convenient if sensitivities with respect to a density field can be calculated. Note that only elements with intermediate density values have an influence on the boundary, and will thus contribute to the sensitivities.

After the density fields for each feature *f* have been created, denoted here as $^{f}\! \mathbf {\bar{x}}$, the steps toward the final component design can be taken. In order to exclude unnecessary features, every $^{f}\! \mathbf {\bar{x}}$ is multiplied with an opacity design variable $^{f}\! \alpha$, similar to Norato et al. (2015). Next, multiple features can be combined with a smooth maximum operation over the feature density fields, which will be applied in Sect. 3.2. This is done through a P-norm:

9 $$\begin{aligned} \hat{x}_{e} = \left( \sum ^{N_{f}}_{f=1}( ^{f}\! \alpha \cdot ^{f}\! \bar{x}_{e})^{P_{1}} \right) ^{\frac{1}{P_{1}}}. \end{aligned}$$

Here, $P_{1}>0$ is the aggregation parameter, and $N_{f}$ is the number of features considered. In this smooth maximum operation overlapping features can lead to output values higher than 1, these are normalized with a nonsmooth maximum operation.

It is also possible to combine a feature with a classical density design variable field $\textbf{x}$, which will be used in Sect. 3.3:

10 $$\begin{aligned} \hat{x}_{e} = \left( ^{f}\! \bar{x}_{e}^{P_{1}} + x_{e}^{P_{1}} \right) ^{\frac{1}{P_{1}}}. \end{aligned}$$

The sensitivities from Eqs. (9) and (10) and follow naturally, and with the chain rule the sensitivities with respect to the design variables can be obtained.

### Regulation of feature shapes

The method described in Sect. 2.1 and 2.2 succeeds in creating features, whose shape can be optimized. However, it was found that undesired feature shapes can emerge during the optimization process. In this section, we will describe these, and some techniques which we use to regulate feature shapes.

Four problems will be addressed: self-intersecting boundaries, uneven PP distribution, proximity of solid and void PPs, and initial design influence. Note that, the proposed restrictions not always address the fundamental cause of a problem and instead restrict it indirectly because of simplicity or associated computational cost. Also, extra constraints add extra complexity to the optimization problem limiting design freedom. Possibly future improvements on the method may make these regulations redundant.

**Fig. 9:**
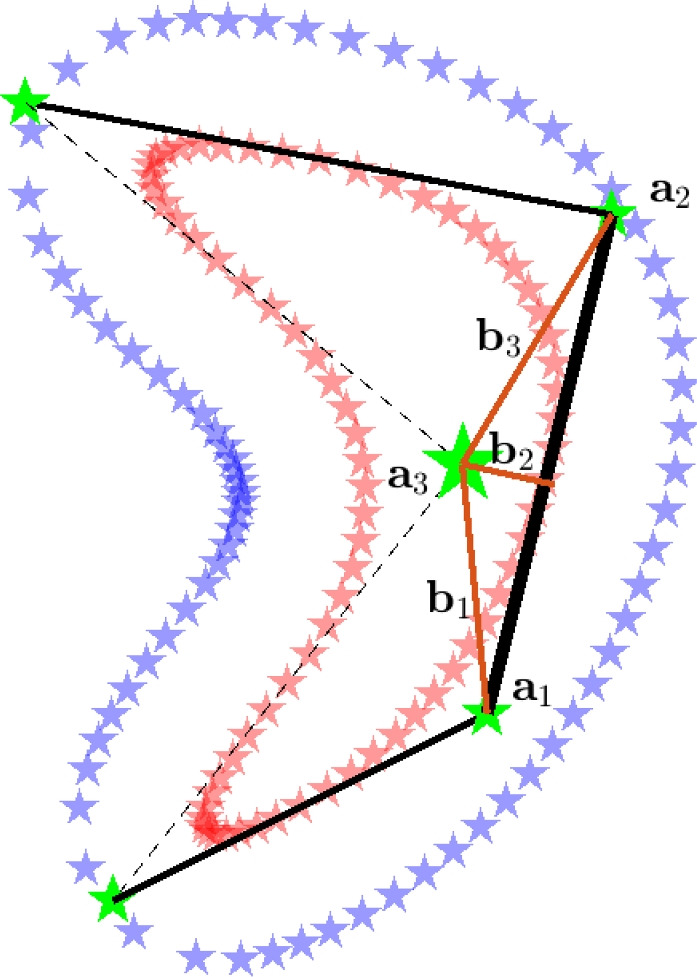
Self-intersecting solid PPs are undesirable. This problem is mitigated by constraining a minimum distance between a CP and line segments between other CPs. For the marked CP ($\textbf{a}_{3}$), the distance is measured toward all black line segments, with the line segment between $\textbf{a}_{1}$ and $\textbf{a}_{2}$ highlighted

The first problem that was tackled, is self-intersecting feature curves. Self-intersecting feature shapes do not necessarily have the intermediate density region on the outside of the feature, while this is crucial for consistent sensitivities. In our application, self-intersecting shapes are prevented by adding a constraint imposed on the minimum distance between a CP and all line segments connecting consecutive CPs. An example is shown in Fig. 9. The signed distance *d* from point $\textbf{a}_{2}$ to the line segment between $\textbf{a}_{1}$ and $\textbf{a}_{3}$ is calculated similarly to, e.g., Smith and Norato (2020) and Norato et al. (2015), and is given by:

11 $$\begin{aligned} \begin{aligned}&d (\textbf{a}_{1},\textbf{a}_{2},\textbf{a}_{3}) := {\left\{ \begin{array}{ll} \Vert \textbf{b}_1 \Vert & \text {if} \quad \textbf{h}_{1} \cdot \textbf{h}_{2} \le 0 , \\ \Vert \textbf{b}_2 \Vert & \text {if} \quad 0< \textbf{h}_{1} \cdot \textbf{h}_{2} < \textbf{h}_{1} \cdot \textbf{h}_{1} , \\ \Vert \textbf{b}_3 \Vert & \text {if} \quad \textbf{h}_{1} \cdot \textbf{h}_{2} \ge \textbf{h}_{1} \cdot \textbf{h}_{1} . \end{array}\right. } \\&\text {with} \quad \textbf{h}_{1} := \textbf{a}_{2} - \textbf{a}_{1} \quad \text {and} \quad \textbf{h}_{2} := \textbf{a}_{3} - \textbf{a}_{1}. \end{aligned} \end{aligned}$$

**Fig. 10:**
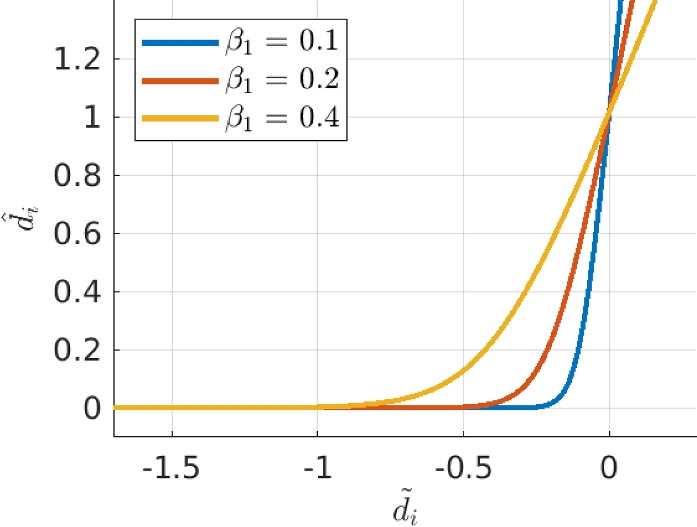
Input is (scaled) distance, output is a constraint value. By adding a maximum constraint value, a minimum distance is ensured strictly

Next, each distance $d_{i}$ between a CP and all line segments connecting the remaining CPs is constrained to stay above a threshold value, $d_{\text {min}}$. First, the distance $d_{i}$ is normalized and transformed to $\tilde{d_{i}}=1-{d_{i}}/{d_{\text {min}}}$, such that $\tilde{d}_{i}>0$ indicates constraint violation, and $\tilde{d}_{i}<0$ indicates a feasible design. Next, a smooth rectifier function is used, which ensures that $\tilde{d}_{i}>0$ values are projected to $\hat{d}_{i}>\epsilon$, while $\tilde{d}_{i}<0$ approach to zero, i.e., $\hat{d}_{i} \rightarrow 0$, including a smooth transition required for the sensitivity analysis. This function is shown in Fig. 10, and it allows for aggregation of all distance constraints through summation. Finally, the inequality constraint for the optimization problem $g_{d}$ is computed as:

12 $$\begin{aligned} \begin{aligned}&\tilde{d}_{i} = 1- \frac{d_{i}}{d_{\text {min}}} \\&\hat{d}_{i} = \frac{ \text {ln} \left( \text {e}^{ \left( \beta _2\frac{ \beta _1 + \tilde{d}_i }{\beta _1} \right) } +1 \right) }{ \beta _2 } . \\&g_{d} = \sum _{i}^{n_{d}} \hat{d}_{i} - \epsilon \\&g_{d} \le 0 \end{aligned} \end{aligned}$$

Here, we use $\beta _1=0.2$ and $\beta _2=3$, $n_{d}$ is the number of line segments considered, and $\epsilon ={ \text {ln} \left( \text {e}^{ \beta _2 } +1 \right) }/{ \beta _2 } \approx 1.016$ is used.

In our experience, this method was found to be simple, fast, and sufficiently effective. However, one downside is that the distance constraint can prevent small features. Also, this method does not prevent self-intersection of the actual curve, since only the zeroth order NURBS curve is constrained, for simplicity. Finally, self-intersection prevention of a zeroth order curve during optimization is also not ensured when the optimization movelimits allow big CP displacements that might lead to CPs jumping over a line segment without violating the constraint.

**Fig. 11:**
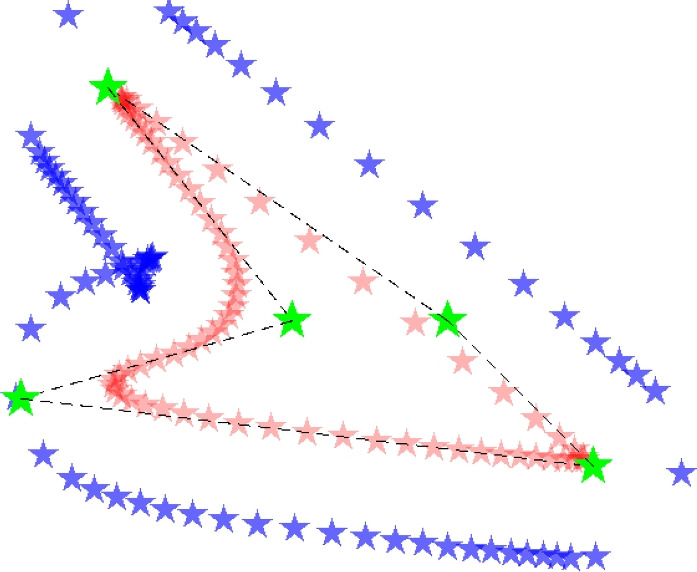
An uneven distribution of void PPs at inner or outer corners is undesirable, for the projection to the density field. This problem is mitigated by restricting the weights of the CPs, and by smoothening of the offset curve calculation

The second problem that has been tackled, is an uneven PP distribution in the void PP offset curve, such as shown in Fig. 11. This occurs especially at sharp corners due to large CP weights. Increasing the number of PPs would mitigate this problem, but with additional computational costs.

Sharp corners can be prevented by limiting the range of $\textbf{p}^{\text {(w)}}$ values. Usually, NURBS weights are allowed within the range [0, 1]. In our implementation a smaller range of [0.25, 0.75] is imposed. Secondly, the distribution of the void PPs is further smoothened through modifying the calculation of the offset curve with $\textbf{n}_{j}$. Instead of using the local normal of the NURBS curve, an approximate normal determined by finite difference using a relatively large step size leads to a more uniform spacing of void PPs. Therefore, in this study, we use a finite difference operation involving PPs $j+2$ and $j-2$:

13 $$\begin{aligned} \begin{aligned}&\textbf{n}_{j} = \frac{\textbf{h}_{1}}{ \Vert \textbf{h}_{1} \Vert }, \\&\text {with} \quad \textbf{h}_{1} := \textbf{h}_{2} \times \textbf{h}_{3}, \\&\text {where} \quad \textbf{h}_{2} := \dot{\textbf{c}}_{j+2} - \dot{\textbf{c}}_{j-2} \quad \text {and} \quad \textbf{h}_{3} := \begin{bmatrix} 0 \\ 0 \\ 1 \end{bmatrix}. \end{aligned} \end{aligned}$$

This is valid for an anticlockwise PP ordering, and since the curve is continuous the first PP of the curve uses the next to last PP of the curve for the $j-2$, and vice versa.

**Fig. 12:**
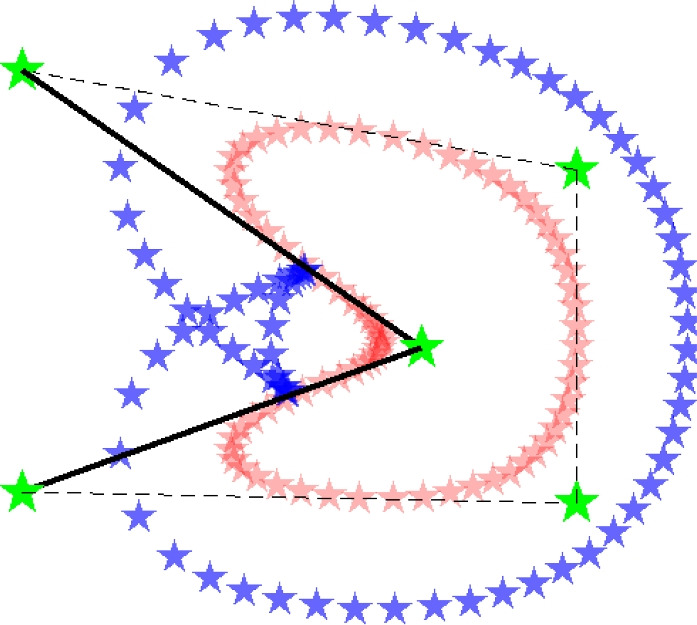
Sharp inner corners can cause an undesirable distribution of void PPs. This problem is mitigated by constraining the angle $\theta$ between connected CPs, with Eq. (14)

The third problem is void PPs getting too close to or inside of the solid PP curve, caused by sharp inner corners. An example of such CP locations is shown in Fig. 12. Therefore, another constraint is added, limiting the angle between two adjacent line segments between CPs. These angles are calculated, and constrained with a similar projection and summation as used before:

14 $$\begin{aligned} \begin{aligned}&\tilde{\theta }_{i} = 1 - \frac{\theta _{i}}{\theta _{\text {min}}} \\&\hat{\theta }_{i} = \frac{ \text {ln} \left( \text {e}^{ \left( \beta _2 \frac{ \beta _1 + \tilde{\theta }_i }{\beta _1} \right) } +1 \right) }{ \beta _2 } . \\&g_{\theta } = \sum _{i}^{n_{\theta }} \hat{\theta }_{i} - \epsilon \\&g_{\theta } \le 0 \end{aligned} \end{aligned}$$

Again, $\beta _1=0.2$ and $\beta _2=3$ are used, $n_{\theta }$ is the number of constrained inner corners, $\epsilon ~=~{ \text {ln} \left( \text {e}^{ \beta _2 } +1 \right) }/{ \beta _2 }$, and the angle $\theta$ is scaled $\tilde{\theta }_{i}~=~1-~{\theta _{i}}/{\theta _{\text {min}}}$, such that the constraint of becomes active for angles smaller than $\theta _{\text {min}}$.

The fourth problem that was tackled concerns the influence of the initial design. It is common in existing LS and FFM methods for the initial design state to have a significant influence on the outcome. Consequently, convergence to different local optima is observed. In our method with fewer features especially, this is relevant. In order to decrease the influence of the initial design, the features are therefore gradually activated with a continuation scheme, similar to the approach presented in van de Ven et al. (2018). In the first iterations of the optimization process, we mix the final feature density field $\mathbf {\hat{x}}$ with a classical density field $\mathbf {{x}}$:

15 $$\begin{aligned} \breve{x}_{e} = (1-\gamma ) {x}_{e} + \gamma \hat{x}_{e}, \end{aligned}$$

where $0 \le \gamma \le 1$ is the mixing parameter. In this paper $\gamma$ is continuously increased from 0 to 1 in the first 20 optimization iterations.

## Numerical examples

In this section, the numerical examples are presented. Section 3.1 presents the optimization formulation and the parameters used. Next, a mechanical optimization problem with solid features is described and analyzed in Sect. 3.2. Subsequently, Sect. 3.3 presents a thermal optimization problem with void features.

### Optimization problem formulation

The first numerical example considers a mechanical optimization of solid features for minimum compliance as objective in the presence of a volume constraint:

16 $$\begin{aligned} \begin{aligned} \underset{\textbf{x}, \textbf{p}}{\text {minimize}} : \quad&C (\breve{\textbf{x}}) = \textbf{u}^{T} \textbf{K}_{m} (\breve{\textbf{x}}) \textbf{u} \\ \text {subject to}: \quad&\textbf{K}_{m} (\breve{\textbf{x}}) \textbf{u} - \textbf{f} = \textbf{0} \\&\frac{V (\breve{\textbf{x}}) }{V^{*}} - 1 \le 0 \\&g_{d} \le 0 \\&g_{\theta } \le 0 \end{aligned} \end{aligned}$$

Here, $\textbf{K}_{m}$, $\textbf{u}$, and $\textbf{f}$ denote the finite element system stiffness matrix, displacement vector, and mechanical load vector. $\textbf{p}$ is used to denote the design variables $\textbf{p}^{\text {(x)}}$, $\textbf{p}^{\text {(y)}}$, and $\textbf{p}^{\text {(w)}}$. Note that $\breve{\textbf{x}} = \breve{\textbf{x}}(\textbf{x}, \textbf{p})$. The objective is compliance *C*, the current design volume is *V*, and the maximum allowed volume is $V^{*}=0.2$ of the design domain.

The second numerical example considers a thermal optimization problem with a single void feature, with minimum thermal compliance as objective and a volume constraint. In this problem, the structure is formed by a density design variable field, combined with a void feature that has a minimum volume. This is done with a minimum operation for Eq. (10), with $P_{1}=-6$. One application for this problem would for example be the placement and shape determination of a tank with a specified minimum volume in a design area. The second optimization problem is given as follows:

17 $$\begin{aligned} \begin{aligned} \underset{\textbf{x}, \textbf{p}}{\text {minimize}} : \quad&C (\breve{\textbf{x}}) = \textbf{T}^{T} \textbf{K}_{t} (\breve{\textbf{x}}) \textbf{T} \\ \text {subject to}: \quad&\textbf{K}_{t} (\breve{\textbf{x}})\textbf{T} - \textbf{q} = \textbf{0} \\&\frac{V(\breve{\textbf{x}})}{V^{*}} - 1 \le 0 \\&g_{d} \le 0 \\&g_{\theta } \le 0 \\&1 - \frac{V_{f}}{V_{f}^{*}} \le 0 \\ \end{aligned} \end{aligned}$$

Now, $\textbf{K}_{t}$, $\textbf{T}$, and $\textbf{q}$ denote the finite element system conductivity matrix, temperature vector, and thermal load vector. The minimum volume constraint of the void feature is added with its current volume denoted by $V_{f}$ and its minimum volume by $V_{f}^{*}$.

For each element *e* the Young’s modulus required for the calculation of $\textbf{K}_{m}$ in Eq. (16) and the conductivity required for the calculation of $\textbf{K}_{t}$ in Eq. (17), the modified SIMP interpolation scheme proposed by Sigmund (2007) is used, i.e.,

18 $$\begin{aligned} E(\breve{x}_{e}) = E_{\text {min}} + \breve{x}_{e}^{p} (E_{\text {max}} - E_{\text {min}}), \end{aligned}$$

with $p=3.0$, and Young’s moduli $E_{\text {min}}=10^{\text {-9}}$ and $E_{\text {max}}=1$ for the mechanical problem. The same interpolation scheme is used for the thermal problem, with conductivities $E_{\text {min}}=10^{\text {-3}}$ and $E_{\text {max}}=1$. For the finite element analysis, a structured mesh comprising 4-node quadrilateral elements with bilinear shape functions is employed. These aspects of the problem are kept relatively simple and standard, as our focus is on evaluating the strengths and weaknesses of the novel formulation.

The problem is implemented as an extension to the 88 line MATLAB code by Andreassen et al. (2011), supplemented with the MMA optimizer by Svanberg (1987) with an additional set of CP design variables. The optimization is terminated after 250 iterations, by which a desired level of convergence was always reached.

Solid features with 8 CPs were used for the mechanical problem, and one void feature with 8, 12, or 16 CPs was used in the thermal problem. Second order basis functions were used for the NURBS. The knot vector is unclamped uniform. The NURBS are discretized with 30 solid PPs per CP. The projection radius *R* is 4.5 times element length $l_{x}$, which is also equal to the separation between solid and void PPs, *r*. The MMA limits for the CP coordinates $\textbf{p}^{\text {(x)}}$ and $\textbf{p}^{\text {(y)}}$ are [0, 1], for a unit square design domain, to keep the CPs inside. The MMA limits for the CP weights $\textbf{p}^{\text {(w)}}$ are [0.25, 0.75] as explained in Sect. 2.3. The minimum distance and angle constraints are set at $d_{\text {min}}=4.5l_{x}$ and $\theta _{\text {min}}=45^\circ$, respectively. An overview of the used parameters is given in Table 1.

**Table 1:** Summary of used parameter values

Parameter	Value
Projection radius *R*	4.5$l_{x}$
Offset curve distance *r*	4.5$l_{x}$
$P_{1}$	6/$-6$
SIMP exponent *p*	3.0
$\hbox {E}_{\text {min}}$	$10^{\text {-3}}$/$10^{\text {-9}}$
$\hbox {E}_{\text {max}}$	1
Poisson’s ratio $\nu$	0.3
Number of iterations	250
Number of solid PPs per CP $n^{\text {(CP)}}$	30
Minimum distance $d_{\text {min}}$	4.5$l_{x}$
Minimum angle $\theta _{\text {min}}$	$45^\circ$

### Mechanical problem with solid features

**Fig. 13:**
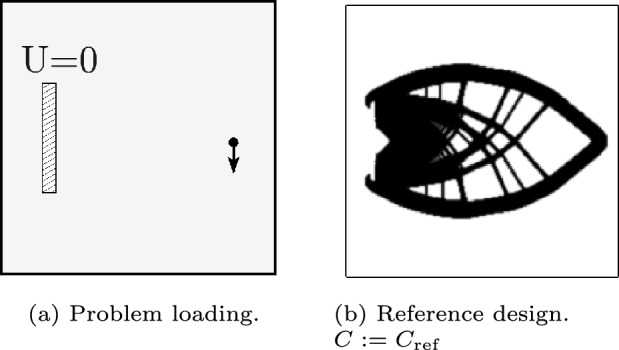
The cantilever beam compliance problem. **a** The load and boundary conditions are shown. The unit load is applied 5% from the right edge, and 50% from the bottom. The fully clamped region is located 10% from the left edge, 50% from the bottom, and has a width of 2% and a height of 30%. **b** An optimization result obtained using the standard density method is shown

The first numerical example is a cantilever beam case. Since this problem has predictable behavior, the performance of the proposed method can be easily observed. The loading, boundary conditions and a conventional density-based TO result are shown in Fig. 13. A discretization of 200$\times$200 elements is used.

**Fig. 14:**
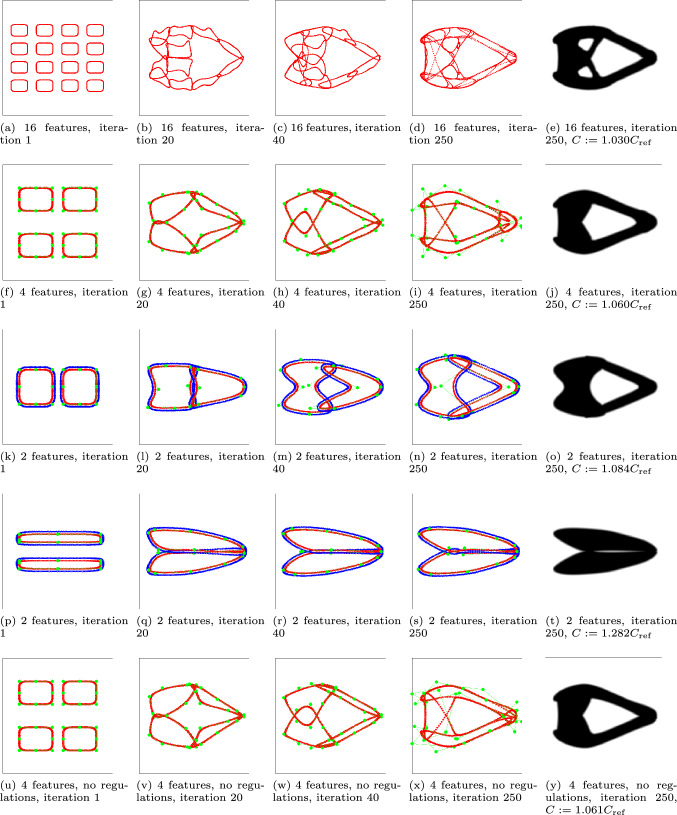
Results of the cantilever beam problem, with 16, 4, and 2 features given in the first, second, and third row, respectively. The fourth row is with 2 features with a different initialization, while the fifth row is with 4 features and no regulations. For clarity, only features with $^{f}\! \alpha> 0.5$ are shown, void PPs have been omitted from cases with more than two features, and CPs have been omitted from the case with more than 4 features

For this load case given in Fig. 13a, the novel method is tested with a different number of features. Initially, as many as 16 features are introduced, similar to what is typically considered in existing FMMs (Wein et al. 2020). Next, the number of features is reduced to 4 and 2, to demonstrate the full potential of the highly flexible feature shapes. The initial feature designs are shown in Figs. 14a, f, and k. Furthermore, optimization with 2 features with another initialization is also performed (Fig. 14p). Finally, the 4 feature optimization is performed without the distance and angle constraints, and without tight movelimits for the CP weights to eliminate sharp corners. However, the mixing of the designs in Eq. (15) is still included to diminish the influence of the initialization.

Several intermediate designs and the end results of the optimizations are shown in Fig. 14. For the first three tests with 16, 4, and 2 solid features, a relatively simple cantilever beam is created. The case with 16 features is very similar to classic FFM, where many features are successfully combined, however the shapes of the individual features are fairly simple. The cases with 4 and 2 features create similar designs with fewer features, utilizing the flexibility in the feature shape of the proposed method. This simplicity comes with only a minor increase in relative compliance, with values of 1.030, 1.060, and 1.084, for the 16, 4, and 2 features, respectively.

The test with an alternative initial design (Fig. 14p-t) shows a similar part shape but the hole in the middle of the structure is absent, and the relative compliance increases considerably to 1.282. The last test shows that, for this load case, a design of almost identical performance can be successfully obtained even without the regularization schemes introduced in Sect. 2.3. However, the proposed regularizations were generally found to further ensure a stable optimization process and mitigate the risk of undesired designs.

**Fig. 15:**
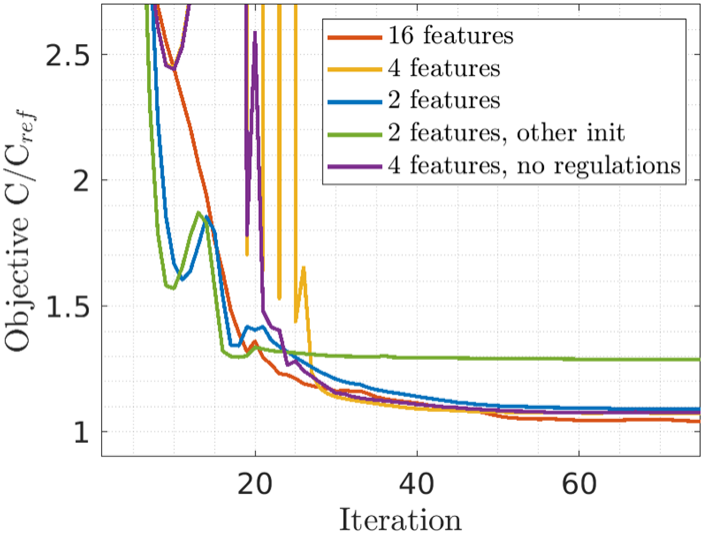
Convergence behaviors for the objective, for all mechanical problems

**Fig. 16:**
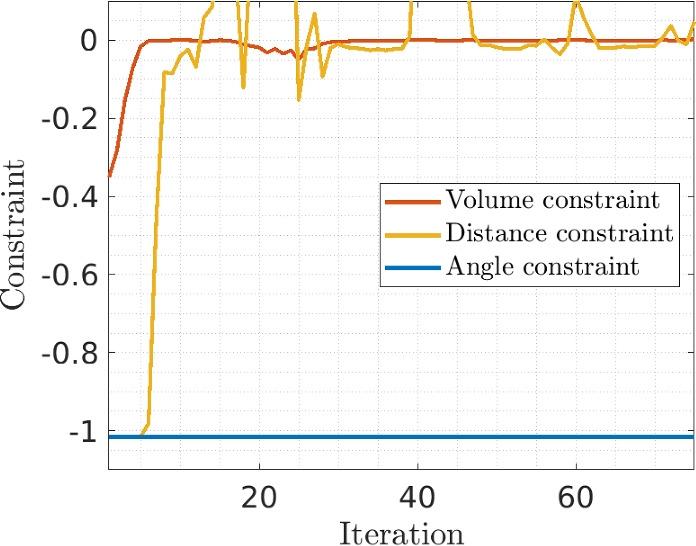
Convergence behavior for the three constraints, for the test with 4 features (second row in Fig. 14)

Next, the convergence performance of the method is considered. Two convergence plots are displayed in Figs. 15 and 16. Irrespective of the number of features considered, swift convergence for the objective is observed, in which the features quickly connect, and smoothly evolve to an optimized shape. Some peaks can be observed until the 20 th iteration, during which the features’ density fields are still combined with a classical density field, leading to a decrease in the objective. However, as the fraction of the classical density field is continuously decreased, the objective can increase. In these three tests, the features are connected around iteration 20, but in our experience features could often still connect at a later stage if not yet connected at iteration 20.

The constraints also show good convergence behavior. The results shown in Fig. 16 for the case with 4 features are representative for other cases. The distance constraint is active or close to be active, which performs as intended. The inner corner angle constraint is not active at any iteration, and would thus not be needed in this case.

### Heat conduction problem with void feature

**Fig. 17:**
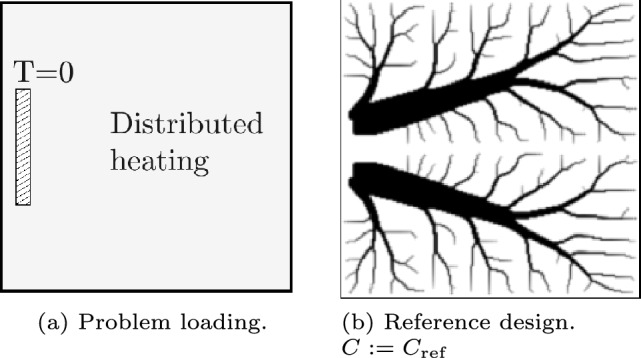
The heat conduction problem. **a** The load and boundary conditions are shown. The heat sink region is located 2.5% from the left edge, 50% from the bottom, and has a width of 5% and a height of 20%. **b** The optimization result without any features is shown as reference

The second numerical example is the heat sink problem, for which the loading condition and a typical conventional density-based TO result are shown in Fig. 17. Again, a discretization of 200$\times$200 is used.

For this case we aim to add a void feature with a specified minimum area, which could for example represent a situation of how and where to locate a (fluid) tank with a flexible shape inside a domain. This is similar to a flexible void area considered in, e.g., Clausen et al. (2014), however with a fixed topology. This design challenge is here combined with the thermal compliance problem, which tends to form a branching network of conductive material throughout the domain (see Fig. 17b). In this way, the void fluid tank represents a conflict for the thermal compliance objective. The void feature field has a value of 1 outside of the feature, and 0 inside of the feature shape. This field is added with a smooth minimum operation to a classical density field. Its opacity parameter is fixed at $\alpha =1$, since the only feature should not disappear.

**Fig. 18:**
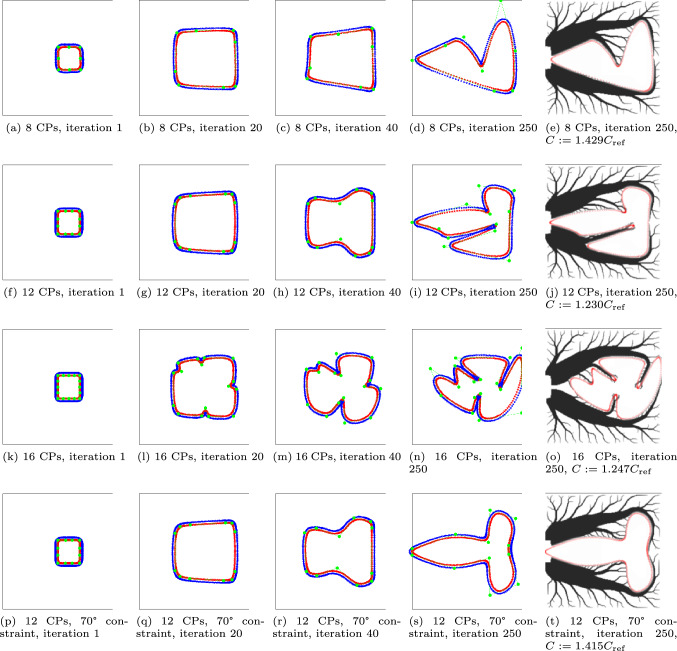
Results of the thermal problem. The first row is one NURBS feature with 8 CPs, the second row with 12 CPs, the third row with 16 CPs, and the fourth row with 12 CPs and an angle constraint of $70^\circ$

First, to study the influence of the number of CPs, the feature is created with 8, 12, or 16 CPs, and the (asymmetric) initial designs can be seen in Fig. 18a, f, and k. Secondly, a 12 CP feature is considered again, but the angle constraint is made more strict to enforce wider inner angles of $70^\circ$ specifically.

The results of the optimizations are shown in Fig. 18. The novel method allows for the void region have a flexible shape, yet it is ensured to keep its topology. The void features with 8, 12, or 16 CPs lead to a relative compliance of 1.429, 1.230, and 1.247, respectively. Increasing the number of CPs increases the shape freedom, however as seen in, e.g., Figure 18n, the void PPs almost penetrate the solid PP curve. This implies the constraints are successful in keeping realistic feature shapes. The feature with 16 CPs can still create sharp inner corners by putting two CPs close next to each other. The minimum distance constraint however still ensures that the void PPs do not touch the solid PP curve.

The fourth case, with a more strict angle constraint, for which the results can be seen in Fig. 18p–t, shows that the constraints succeed in controlling the feature shape and a more rounded void region is obtained albeit with a relative compliance of 1.415.

**Fig. 19:**
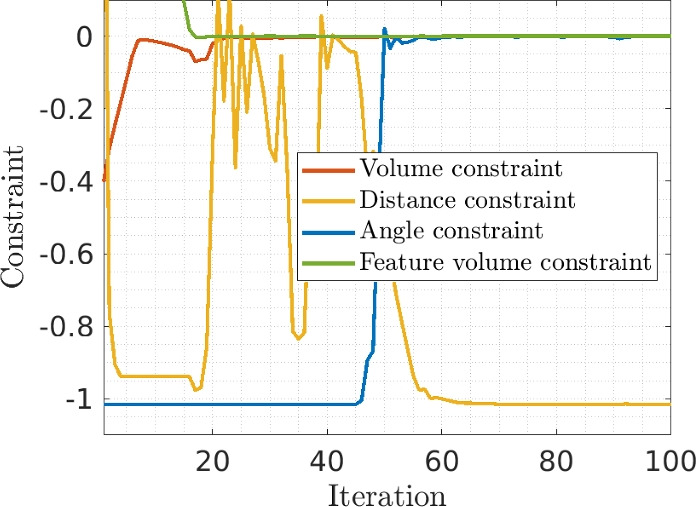
Convergence behavior for the three constraints, for the thermal test with 12 CPs (second row in Fig. 18)

A constraint convergence plot for the thermal problem problem is given in Fig. 19. As can be seen, all constraints except the distance constraint are active or close to be active. For this problem all constraints are needed in keeping a desired feature shape.

## Discussion

As shown in Sect. 3, the proposed method succeeds in optimizing feature shapes with high geometric flexibility. This new method offers a combination of flexibility and control, which is different from more restricted explicit methods yet more flexible than existing implicit feature-based methods. Next to these advantages, the method however still has some shortcomings. The nature of the method has two major downsides: (i) the high feature flexibility necessitates applying restrictions on flexible shaped features in general (ii) our approach for obtaining sensitivities, using a void PP offset curve.

First, the feature shape freedom of the novel FFM method can be misused to create unrealistic and undesired flexible structures, e.g., self-intersection. The restrictions proposed in Sect. 2.3 aim to regulate the shape freedom, in order to only create realistic features, even though the regulations also limit the design freedom and may prohibit certain admissible shapes. The problem of how much shape restriction is required, is inherent to shaped features, and becomes particularly evident with the relatively high shape flexibility introduced by our method. While the methods we have developed proved effective, it is quite possible that less restrictive and less conservative regularization formulations are possible. Furthermore, the concept of flexible features regulated with various restrictions/constraints also offers new opportunities: for specific ranges of shape deformations specific geometric rules could be imposed, allowing, e.g., combining parametric design with TO. Both aspects are identified as directions for future research.

Secondly, the void PP offset curve, an essential part for the gray boundary, which is crucial for obtaining sensitivities, introduces challenges. For example, in the presented formulation the void PP curve can suffer from uneven PP distributions with big gaps or clustering, or void PPs can penetrate the solid PP curve. One advantage advocating for the offset curve is consistent sensitivities in void regions surrounded by solid regions, such that this location is equally close to multiple boundaries/edges.

Next, the feasibility of extending the method to 3D should be addressed. For all operations in Sect. 2.1 and 2.2 the extensions to a third dimension follow straightforward. However, the feature shape problems addressed in Sect. 2.3 could potentially be more challenging. Naturally, 3D features are created with more CPs, which increases the design freedom, with potential for unrealistic shapes. Furthermore, the presented regulation methods do not directly transfer to 3D. The minimum distance calculation from Eq. (11) should become a distance between a CP and a plane segment. Similarly, the angle used in Eq. (14) should potentially be between several neighboring CPs, or a different way to smoothen the normals should be applied. While these are definitely challenges that require further investigation, the concepts introduced by the proposed method fundamentally carry over to 3D.

With the expansion to 3D, further research can be done on controlling feature sizes. This is a useful property of explicit FFMs, and integrating it with the proposed method could further benefit practical applications.

In our experience using an unoptimized implementation, the computational cost of all feature operations (creation and sensitivities) was less than 10% of the FE solve, in the studied problems. Feature operation costs scale up linearly with the number of features, and the number of PPs, so the fraction of the feature operations on the total cost will be even lower for problems involving finer meshes. It is important to note that our approach requires a limited number of control points to describe complex feature shapes which is favorable for computational tractability.

Ultimately, FFMs are methods in which feature boundaries move. Just like in LS methods, but with shape restrictions to ensure simpler designs. Consequently, design outcomes are limited to a subset of all possible geometries, which likely also implies reduced performance. However, the degree of restriction is controlled by the designer. Our method provides a new option to control the range from full design freedom and potentially complex outcomes, to limited design freedom and simple outcomes. For each specific combination of application demands, manufacturing and cost considerations it allows for a desired amount of restrictions and regulations.

## Conclusion

A new method for feature optimization with flexible feature shapes is proposed, through defining feature shapes with NURBS. With the NURBS control points and their weights as design variables a feature density field is constructed. The method builds on conventional density-based TO procedures. All steps are differentiable, allowing for consistent sensitivities to be calculated.

Features can be created with complex shapes, allowing for layouts to be represented with a fixed topology, by fewer features, and with varying degrees of geometric control. The features can be either solid or void, including an opacity parameter, and used seamlessly in combination with established density methods. As shown by different numerical examples, the method succeeds in optimizing feature shapes with good convergence characteristics, and follows for solving new problems with a degree of shape control not available in other approaches.

In several aspects, the proposed method offers potential for further development. Firstly, extending its implementation to 3D is worth exploring. While the concept fundamentally remains the same as in the planar case, various implementation details may need additional consideration. Secondly, to enable specific control of feature shapes, the set of regulation schemes proposed and demonstrated in this paper could be further refined and extended. Along this path the method offers a way to combine parametric design concepts and free-form TO, and provides designers with new means to control the computational design process.

## Data Availability

The necessary information for replication of the results is present in the manuscript. To help readers reproduce results, further algorithm details are provided on reasonable request.
